# Reproductive characteristics are associated with gene-specific promoter methylation status in breast cancer

**DOI:** 10.1186/s12885-019-6120-4

**Published:** 2019-09-18

**Authors:** Lauren E. McCullough, Lindsay J. Collin, Kathleen Conway, Alexandra J. White, Yoon Hee Cho, Sumitra Shantakumar, Mary Beth Terry, Susan L. Teitelbaum, Alfred I. Neugut, Regina M. Santella, Jia Chen, Marilie D. Gammon

**Affiliations:** 1 Department of Epidemiology, Emory University, Atlanta, GA 30322 USA; 2 Department of Epidemiology, University of North Carolina, Chapel Hill, NC 27599 USA; 3 Epidemiology Branch, National Institute of Environmental Health Science, Research Triangle Park, NC 27709 USA; 4 Department of Biomedical and Pharmaceutical Sciences, University of Montana, Missoula, MT 59812 USA; 5 Epidemiology, Real World Evidence and Digital Platforms, Glaxosmithkline, Singapore, Singapore; 6 Department of Epidemiology, Columbia University, New York, NY 10032 USA; 7 Department of Medicine, Columbia University, New York, NY 10032 USA; 8 Department of Environmental Health Sciences, Columbia University, New York, NY 10032 USA; 9 Department of Environmental Medicine and Public Health, Icahn School of Medicine at Mount Sinai, New York, NY 10029 USA; 10 Department of Pediatrics, Icahn School of Medicine at Mount Sinai, New York, NY 10029 USA; 11 Department of Oncological Science, Icahn School of Medicine at Mount Sinai, New York, NY 10029 USA

**Keywords:** Breast Cancer, Epidemiology, Epigenetics, Reproductive characteristics, DNA methylation

## Abstract

**Background:**

Reproductive characteristics are well-established risk factors for breast cancer, but the underlying mechanisms are not fully resolved. We hypothesized that altered DNA methylation, measured in tumor tissue, could act in concert with reproductive factors to impact breast carcinogenesis.

**Methods:**

Among a population-based sample of women newly diagnosed with first primary breast cancer, reproductive history was assessed using a life-course calendar approach in an interviewer-administered questionnaire. Methylation-specific polymerase chain reaction and Methyl Light assays were used to assess gene promotor methylation status (methylated vs. unmethylated) for 13 breast cancer-related genes in archived breast tumor tissue. We used case-case unconditional logistic regression to estimate adjusted odds ratios (ORs) and 95% confidence intervals (CIs) for associations with age at menarche and parity (among 855 women), and age at first birth and lactation (among a subset of 736 parous women) in association with methylation status.

**Results:**

Age at first birth > 27 years, compared with < 23 years, was associated with lower odds of methylation of *CDH1* (OR = 0.44, 95% CI = 0.20–0.99) and *TWIST1* (OR = 0.48, 95% CI = 0.28–0.82), and higher odds of methylation of *BRCA1* (OR = 1.63, 95% CI = 1.14–2.35). Any vs. no lactation was associated with higher odds of methylation of the *PGR* gene promoter (OR = 1.59, 95% CI = 1.01–2.49). No associations were noted for parity and methylation in any of the genes assayed.

**Conclusions:**

Our findings indicate that age at first birth, lactation and, perhaps age at menarche, are associated with gene promoter methylation in breast cancer, and should be confirmed in larger studies with robust gene coverage.

## Background

Breast development is a complex biological process that occurs in several phases across the life-course; initiated during the embryonic period, continuing through puberty, with terminal differentiation following first birth and lactation. [1] Consistent with mammogenesis, there is accumulating evidence that early life characteristics play an important role in the etiology of breast carcinogenesis. [2] Reproductive characteristics that contribute to cumulative hormonal exposure, such as age at menarche, parity, age at first birth, and lactation, are well-established risk factors for breast cancer. [3] However, the mechanisms underlying these associations remain unresolved.

We hypothesized that reproductive characteristics could potentially be differentially associated with breast cancer based on epigenetic alterations in the tumor. Aberrant DNA methylation, an epigenetic modification, can modify gene expression to impact breast carcinogenesis. [4, 5] For example, promoter hypermethylation of tumor suppressor genes has been associated with clinical and pathological factors of breast cancer, as well as mortality in a population-based sample. [6] DNA methylation alterations are associated with environmental and lifestyle factors and could be a biologic mechanism for disease. [7] Tobacco smoke, nutrient intake, and air pollution exposure are all associated with epigenetic modification through gene promoter methylation. [8] The association between epigenetic modifications and reproductive characteristics has received limited attention. In one previous study of 803 archived breast tumors, no differences in promoter methylation of *E-cadherin (CDH1)*, *CDKN2A,* or *RAR-β2* by age at menarche or age at first birth were noted. [9] However, this previous research was limited to only three breast cancer-related genes and did not consider associations with parity or lactation.

To address our hypothesis, we examined whether four reproductive characteristics (age at menarche, age at first birth, lactation, and parity) were associated with promoter methylation status in a panel of 13-breast cancer-related genes measured in archived tumor tissue of a population-based sample of women with newly diagnosed breast cancer.

## Methods

We utilized resources from case women enrolled in the Long Island Breast Cancer Study Project (LIBCSP), a population-based study. [10] Institutional Review Board approval was obtained by all participating institutions (Columbia University, University of North Carolina Chapel Hill, and Emory University).

### Study population

Study participants were residents of Nassau and Suffolk counties, Long Island, New York (NY). Eligible case women were diagnosed with first primary breast cancer between August 1, 1996 and July 31, 1997 identified using rapid case ascertainment via daily or weekly contact with pathology departments of all 28 hospitals on Long Island, and three tertiary care hospitals in NY City. [10] At the time of diagnosis, women were aged 20–98 years (67% postmenopausal) and primarily white (94%). [10]

### Reproductive and covariate assessment

Interviews for most participants occurred within 3 months of diagnosis (before completion of the first course of treatment) [10] and were completed for 82.1% (*N* = 1508) of eligible women. Written informed consent was obtained from all women prior to study interview.

Reproductive characteristics (occurring prior to the date of diagnosis) were assessed as part of the 100-min, in-home, interviewer-administered questionnaire. To aid recall, a month-by-month calendar approach [11] was used to record reproductive factors in the context of major life events. Age at menarche (≤12 vs. > 12 years of age), age at first birth among parous women (< 23, 23–27, > 27 years of age), lactation practices among parous women (ever vs never), and parity (nulliparous vs parous), were assessed in these analyses. Category cut points were based on previous literature [12] and optimization of LIBCSP cell counts.

Women were additionally asked about their: demographic characteristics; lifestyle, environmental, and medical histories; family history of breast cancer; as well as use of exogenous hormones. [10]

### Gene-specific promoter DNA methylation assessment

Archived pathology blocks were obtained from the participating hospitals for 962 (63.8%) case participants; [13] tumor tissue was available for 855 (56.7%) women. As previously described, promoter methylation status was measured in tumor tissue for a panel of 13 breast cancer-related genes [adenomatous polyposis coli (*APC*), breast cancer 1, early onset (*BRCA1*), cyclin D2 (*CCND2*), E-Cadherin (*CDH1*), death-associated protein kinase 1 (*DAPK1*), estrogen receptor 1 (*ESR1*), glutathione S-transferase pi 1 (*GSTP1*), secretoglobin, family 3A, member 1 (*HIN1*), cyclin-dependent kinase inhibitor 2A (*CDKN2A*), progesterone receptor (*PGR*), retinoic acid receptor beta (*RARβ*), Ras association domain family member 1 (*RASSF1A*) and twist homolog 1 (*TWIST1*)]. [14] While a broader panel could be hypothesis generating, given the sample size, a more focused panel of genes reduces chances of type II error. These genes were selected based on their putative functions and their promoter regions are frequently methylated in breast tumor tissues. [14]

For study participants with available tissue blocks, the paraffin blocks were used to generate 15 × 5 micron and 100 μm this slides, which were isolated via microdissection. Tumour DNA was isolated by adding 30 ul of proteinase K-digestion buffer and with overnight incubation. After DNA extraction from the archived tumor tissue, [15] gene-specific promoter methylation status was assessed for 13 genes. [14, 16] Promoter methylation of *ESR1*, *PGR* and *BRCA1* was determined by methylation-specific (MSP) polymerase chain reaction (PCR), as described previously. [15, 17] For select genes (*ESR1*, *PGR* and *BRCA1*), the methylation status was determined by whether PCR product was obtained using methylation-specific primers—thus, are dichotomous variables (methylated vs. unmethylated). The quantitative MethyLight assay was used to determine methylation status of the remaining 10 genes. Bisulfite-converted genomic DNA was amplified using a fluorescence-based, real-time quantitative PCR, which yields a continuous measure of methylation. [18] Percentage of methylation was calculated by the 2^-ΔΔCT^ method, where ΔΔC_T_ = (C_T,Target_ - C_T,Actin_)_sample_ - (C_T,Target_ - C_T,Actin_)_fully methylated DNA_ [19] and multiplying by 100. For consistency with previous published reports by our study team [14] and others, we dichotomized (< 4%, ≥4% methylated) the resulting values, [20] as it has been previously shown to distinguish between malignant and normal tissues and is indicative of repressed gene expression. [21] The numbers of assayed samples and corresponding methylation frequencies for the selected genes are summarized in Xu et al. [14] Insufficient DNA, primarily due to small tumor size, was the primary main reason for missing methylation data.

### Hormone receptor (HR) subtype assessment

Breast cancer subtype for the first primary was defined by estrogen/progesterone receptor status (ER/PR) obtained from the medical record, and was available for 65.6% of cases (*N* = 990). [10] ER/PR and tumor methylation status were available for 63.3% (*N* = 627) of our participants. Given that reproductive characteristics have been etiologically linked to breast cancer primarily through an estrogen pathway, we did not consider human epidermal growth factor receptor 2 (HER2) in our subtype assessment.

### Statistical methods

All statistical analyses were conducted using SAS 9.4 (Cary, NC) using a two sided *p*-value < 0.05 as the cutoff for statistical significance. Employing a case-case approach, we assessed whether four reproductive characteristics (considered independently) were associated with methylation in tumor tissue. We used unconditional logistic regression [22] to estimate odds ratios (ORs), and corresponding 95% confidence intervals (CIs) for each of the 13 markers, with case groups characterized by tumor methylation status (methylated vs. unmethylated). For age at menarche and parity, models included all cases with tumor tissue available (*N* = 855); for age at first birth and lactation, models were restricted to parous women only (*N* = 736). The case-case OR estimates the likelihood of a case possessing a methylated gene-promoter given their specific reproductive characteristic. ORs greater than 1 indicate higher odds of methylation, while ORs less than 1 indicate lower odds of methylation.

Given reproductive characteristics likely influence breast carcinogenesis through an estrogen pathway, [23, 24] we explored whether the association between reproductive characteristics and hormone receptor status (ER + PR+ vs. all others: ER-PR-, ER + PR-, ER-PR+) varied by gene-specific promoter methylation. We used unconditional logistic regression to estimate ORs (95% CIs) where the OR estimates the likelihood of an ER + PR+ case, given both gene methylation and reproductive characteristics. Using a likelihood ratio test, we assessed evidence for multiplicative interaction—comparing multivariable models with and without cross-product terms to represent the interaction between reproductive characteristics and a gene-specific methylation marker (α = 0.05). A significant interaction would suggest that the odds of a case possessing the ER + PR+ breast cancer subtype, given the reproductive characteristic, are statistically different across strata of gene-specific methylation.

Confounders were identified based on the known epidemiology of breast cancer and analysis of causal diagrams (DAG). [25] For all models, DAG-identified confounders included: race (white/black/other); family history of breast cancer (yes/no); and history of benign breast disease (yes/no), and 5-year age group. Confounders were included in the model if their removal changed the effect estimate > 10%. [26] Only 5-year age group remained in the final case-case models. We did not consider simultaneous adjustment of reproductive factors because they did not meet the causal structure of a confounder and, were highly correlated.

## Results

The distribution of demographic and clinical/pathological characteristics among cases with any tumor methylation marker (*N* = 855) were similar to the corresponding distributions among all LIBCSP participants with breast cancer (*N* = 1508) (Table 1).

**Table 1:** Distribution of reproductive and tumor characteristics among case women with any methylation marker (*N* = 855) and among all case women in a population-based study (*N* = 1508), Long Island Breast Cancer Study Project

	Patients with any methylation marker	All patients
	(Total *N* = 855)	(Total *N* = 1508)
	N (%)	N (%)
Age at diagnosis		
< 50 years	215 (25.1)	407 (27.0)
≥ 50 years	640 (74.9)	1101 (73.0)
Menopausal status		
premenopausal	244 (29.2)	472 (31.9)
postmenopausal	593 (70.8)	1006 (68.1)
missing	18	30
Age at Menarche		
≤ 12 years	372 (44.0)	658 (44.0)
> 12 years	473 (56.0)	837 (56.0)
missing	10	13
Parity		
Nulliparous	119 (13.9)	198 (13.1)
Parous	736 (86.1)	1310 (86.9)
Age at First Birth (among parous women only)		
≤ 23 years	242 (32.9)	437 (33.4)
23–27 years	241 (32.7)	430 (32.9)
≥ 27 years	253 (34.4)	442 (33.8)
missing	0	1
Lactaction (among parous women only)		
Never	462 (62.8)	830 (63.4)
Ever	274 (37.2)	480 (36.6)
Tumor Stage		
In Situ	104 (12.2)	235 (15.6)
Invasive	751 (87.8)	1273 (84.4)
ER Status		
Positive	478 (76.2)	726 (73.3)
Negative	149 (23.8)	264 (26.7)
missing	228	518
PR Status		
Positive	399 (63.6)	635 (64.1)
Negative	228 (36.3)	355 (35.9)
missing	228	518

Estimates for the associations between each of the four individual reproductive characteristics and the 13 gene-specific methylation markers are shown in Tables 2–5. We observed an inverse association between age at menarche ≤12 years (vs. > 12 years) and methylation of the *BRCA1* promoter (OR = 0.79, 95% CI = 0.60–1.04) (Table 2). Conversely, late age at first birth (> 27 years vs. < 23 years) was associated with increased odds of methylation of *BRCA1* (OR = 1.63, 95% CI = 1.14–2.35) and lower odds of methylation at *CDH1* (OR = 0.44, 95% CI = 0.20–0.99) and *TWIST1* (OR = 0.48, 95% CI = 0.28–0.82) gene promoters (Table 3). Any vs. no lactation was associated with higher odds methylation of the *PGR* gene promoter (OR = 1.59, 95% CI = 1.01–2.49) (Table 4). We observed no associations between parity and methylation of any of the 13 gene promoters (Table 5).

**Table 2:** Age-adjusted Odds ratios (ORs) and 95% confidence intervals (CIs) for the association between age at menarche and breast cancer defined by gene-specific promoter methylation (comparing case women with and without methylated breast cancer [*N* = 855]), Long Island Breast Cancer Study Project

	Over 12 years of age			12 years of age or younger		
*Gene promoter*	Methylated/Unmethylated	OR	95% CI	Methylated/Unmethylated	OR	95% CI
*APC*	207/235	1.00	reference	177/172	1.17	0.89–1.55
*BRCA1*	290/180	1.00	reference	208/163	0.79	0.60–1.04
*CDH1*	23/403	1.00	reference	20/311	1.13	0.61–2.09
*CCND2*	83/343	1.00	reference	65/266	1.03	0.71–1.48
*DAPK*	61/365	1.00	reference	46/285	0.98	0.65–1.49
*ESR1*	215/251	1.00	reference	162/205	0.92	0.70–1.22
*GSTP1*	115/311	1.00	reference	97/234	1.12	0.82–1.54
*HIN*	268/158	1.00	reference	208/123	1.00	0.74–1.34
*P16*	14/413	1.00	reference	16/325	1.45	0.70–3.02
*PGR*	50/420	1.00	reference	49/322	1.28	0.84–1.94
*RARB*	123/303	1.00	reference	85/246	0.86	0.62–1.18
*RASSF1A*	363/63	1.00	reference	281/50	0.98	0.66–1.47
*TWIST1*	62/364	1.00	reference	53/278	1.13	0.76–1.69

**Table 3:** Age-adjusted Odds ratios (ORs) and 95% confidence intervals (CIs) for the association between age at first birth and breast cancer as defined by gene-specific promoter methylation (comparing parous case women with and without methylated breast cancer [*N* = 736]), Long Island Breast Cancer Study Project

	≤23 years			23–27 years of age			≥27 years		
*Gene promoter*	Methylated/ Unmethylated	OR	95% CI	Methylated/ Unmethylated	OR	95% CI	Methylated/ Unmethylated	OR	95% CI
*APC*	105/120	1.00	reference	108/122	1.08	0.90–1.30	118/115	1.18	0.81–1.70
*BRCA1*	130/110	1.00	reference	135/106	1.28	1.07–1.53	166/86	1.63	1.14–2.35
*CDH1*	20/209	1.00	reference	9/199	0.66	0.44–1.00	10/220	0.44	0.20–0.99
*CCND2*	45/184	1.00	reference	44/164	1.00	0.79–1.26	46/184	1.00	0.63–1.59
*DAPK*	38/191	1.00	reference	32/176	0.78	0.60–1.02	25/205	0.61	0.36–1.04
*ESR1*	108/130	1.00	reference	117/122	0.94	0.79–1.12	105/143	0.88	0.62–1.26
*GSTP1*	67/162	1.00	reference	59/149	0.90	0.74–1.11	58/172	0.82	0.54–1.23
*HIN*	150/79	1.00	reference	129/79	0.87	0.72–1.05	135/95	0.75	0.51–1.09
*P16*	13/205	1.00	reference	7/216	0.69	0.42–1.11	7/219	0.47	0.18–1.24
*PGR*	27/213	1.00	reference	25/216	1.14	0.86–1.49	35/217	1.29	0.75–2.23
*RARB*	59/170	1.00	reference	58/150	1.08	0.88–1.32	66/164	1.16	0.77–1.75
*RASSF1A*	188/41	1.00	reference	178/30	1.21	0.94–1.56	200/30	1.46	0.88–2.44
*TWIST1*	44/185	1.00	reference	30/178	0.69	0.53–0.91	24/206	0.48	0.28–0.82

**Table 4:** Age-adjusted Odds ratios (ORs) and 95% confidence intervals (CIs) for the association between lactation practices and breast cancer as defined by gene-specific promoter methylation in breast tumor tissue (comparing parous case women with and without methylated breast cancer [*N =* 736]), Long Island Breast Cancer Study Project

	Any Lactation			No Lactation		
*Gene promoter*	Methylated/ Unmethylated	OR	95% CI	Methylated/ Unmethylated	OR	95% CI
*APC*	113/146	1.00	reference	218/211	1.33	0.97–1.81
*BRCA1*	166/107	1.00	reference	265/195	0.89	0.66–1.21
*CDH1*	19/228	1.00	reference	20/400	0.60	0.31–1.15
*CCND2*	51/196	1.00	reference	84/336	0.96	0.65–1.42
*DAPK*	41/206	1.00	reference	54/366	0.74	0.47–1.15
*ESR1*	117/151	1.00	reference	213/244	1.13	0.83–1.53
*GSTP1*	71/176	1.00	reference	113/307	0.91	0.64–1.30
*HIN*	160/87	1.00	reference	254/166	0.83	0.60–1.16
*P16*	10/243	1.00	reference	17/397	1.05	0.47–2.34
*PGR*	41/232	1.00	reference	46/414	0.63	0.40–0.99
*RARB*	70/177	1.00	reference	113/307	0.93	0.65–1.32
*RASSF1A*	214/33	1.00	reference	352/68	0.79	0.51–1.25
*TWIST1*	37/210	1.00	reference	61/359	0.96	0.62–1.50

**Table 5:** Age-adjusted Odds ratios (ORs) and 95% confidence intervals (CIs) for the association between parity and breast cancer as defined by gene-specific promoter methylation (comparing case women with and without methylated breast cancer [*N* = 855]), Long Island Breast Cancer Study Project

	Parous			Nulliparous		
*Gene promoter*	Methylated/Unmethylated	OR	95% CI	Methylated/Unmethylated	OR	95% CI
*APC*	331/357	1.00	reference	56/56	1.10	0.74–1.64
*BRCA1*	431/302	1.00	reference	73/45	1.11	0.75–1.66
*CDH1*	39/629	1.00	reference	5/93	0.87	0.33–2.27
*CCND2*	135/532	1.00	reference	15/83	0.75	0.42–1.35
*DAPK*	95/572	1.00	reference	13/85	0.97	0.52–1.82
*ESR1*	330/395	1.00	reference	53/65	0.98	0.66–1.45
*GSTP1*	184/483	1.00	reference	29/69	1.11	0.69–1.76
*HIN*	414/253	1.00	reference	67/31	1.32	0.84–2.08
*P16*	27/640	1.00	reference	3/107	not estimated^a^	
*PGR*	87/646	1.00	reference	15/103	1.07	0.60–1.93
*RARB*	183/484	1.00	reference	28/70	1.08	0.68–1.74
*RASSF1A*	566/101	1.00	reference	86/12	1.34	0.70–2.55
*TWIST1*	98/569	1.00	reference	18/80	1.36	0.78–2.38

^a^Point estimate was not calculated because cell sizes less than five

When we explored ER/PR status of breast cancer in addition to methylation status, early age at menarche was associated with low odds of ER + PR+ breast cancer in the presence of methylated *RASSF1A* (OR = 0.59; 95% CI = 0.40–0.86) (Additional file 1: Table S1), whereas the corresponding OR among women with unmethylated *RASSF1A* was 1.64 (95% CI = 0.67–3.99) (multiplicative *p*_interaction_ = 0.04) (Additional file 1: Table S1). *BRCA1* methylation also modified the association between age at first birth and odds of ER + PR+ breast cancer (Additional file 1: Table S2. The odds of developing ER + PR+ breast cancer was 2.34 (95% CI = 1.18–4.64) among women with late age at first birth (> 27 years) and unmethylated *BRCA1* promoters, whereas among women with methylated *BRCA1* the OR was 0.88 (95% CI = 0.51–1.51). We identified no differential associations by gene promoter methylation status between lactation or parity and ER + PR+ breast cancer (Additional file 1: Tables S3 and S4).

## Discussion

Our study showed that reproductive characteristics, established risk factors for breast cancer, were associated with methylation sites in tumor tissue of women with breast cancer. Our findings lend support to our hypothesis that reproductive characteristics may be differentially associated with breast cancer based on the methylation status of the tumor.

Specifically, we observed higher odds of methylation for *BRCA1* in association with late age at first birth. *BRCA1* is a tumor suppressor gene, and higher odds of methylation levels are associated with reduced expression in The Cancer Genome Atlas (TCGA) data. [27] Conversely, we observed lower odds of tumor methylation of *CDH1* and *TWIST1*, both involved in epithelial-mesenchymal transition (EMT), in association with late age at first birth. E-cadherin protein is encoded by *CDH1* (16q22.1) and mediates hemophilic cell-cell adhesion between neighboring cells. [28] Loss of E-cadherin is considered a fundamental event in EMT, [29] and is associated with invasion and metastasis of breast cancer cells. [30] A priori, we hypothesized that late age at first birth would be associated with higher odds of *CDH1* promoter methylation in breast tumor tissue, thereby resulting in gene silencing and reduced expression of the E-cadherin protein. Our finding of a monotonic reduction in breast tumor *CDH1* methylation with increasing age at first birth is counter to our hypothesis and could be due to chance with less than 10 methylated cases in each age stratum. Further, while DNA methylation of *CDH1* is an important mechanism for inhibition of E-cadherin protein expression in breast cancer cell lines, [31, 32] studies examining methylation of primary breast cancer tissues remain limited and are conflicting. [33, 34]

Twist-related protein 1 is a basic helix-loop-helix transcription factors implicated in cell lineage determination and differentiation. Our observation of reduced methylation of *TWIST1* with late age at first birth is consistent with our hypothesis of oncogenic activation. Overexpression of Twist or methylation of its promoter is common in metastatic carcinomas, including breast. [35] Thus, age at first birth may both increase methylation of oncogenes and repress methylation of tumor suppressor genes, which may have implications for both gene expression and cell functioning.

We also observed that among parous women who did not breastfeed, the odds of methylation of the *PGR* gene promoter in breast cancer was reduced. Decreased expression of *PGR*, a steroid hormone receptor that helps to maintain normal cell growth and regulation, also plays a role in breast carcinogenesis; although links between *PGR* promoter methylation and protein expression are weak and unlikely to represent the predominant mechanism of receptor silencing. [36]

In our population-based sample, we considered gene-specific methylation with reproductive characteristics, and explored heterogeneity by hormone receptor subtype (ER + PR+ vs. all others), as locus-specific methylation may be particularly associated with certain breast cancer tumor subtypes. [37, 38] We found that women with early age at menarche and promoter *RASSF1A* methylation had lower odds of developing ER + PR+ breast cancer than women with unmethylated *RASSF1A* promoters. Ras association domain-containing protein 1 is a protein that, in humans, is encoded by the *RASSF1* gene, a putative tumor suppressor, involved in cell cycle control [14] and breast carcinogenesis. [39] Thus, our findings are contrary to our biologically driven hypothesis of enhanced odds of ER + PR+ breast cancer with early menarche and *RASSF1A* promoter methylation. They further conflict with a previous report of a positive correlation between *RASSF1A* methylation levels and percentage of cancer cells expressing ER and PR. [40]

We also observed that the odds of being an ER + PR+ breast cancer case was enhanced among women with late age at first birth (> 27 years) in the presence of unmethylated *BRCA1* promoter. As described above, *BRCA1* is a tumor suppressor and its methylation has been associated with loss of BRCA1 expression. The triple-negative subtype (ER−/PR−/HER2-) is associated with *BRCA1* germline and somatic mutations [41] and our observation of a more than two-fold increase in odds of ER + PR+ breast cancer (vs. any ER- or PR-) among women jointly characterized as having late age at first pregnancy and unmethylated *BRCA1* promoter is consistent with these findings.

Strengths of our study include our population-based design. This approach enhances generalizability and facilitates quantification of any study bias due to subject selection. We also used a detailed method to assess reproductive characteristics, which reduces the likelihood of measurement error. In addition, our case-case approach rules out differential recall bias given that both the “case” and “comparison” groups had breast cancer. Limitations of our study include that we were unable to obtain archived tumor tissue for all LIBCSP case participants, which may result in selection bias as smaller tumors would be less likely to have sufficient tumor tissue available for the methylation assays. However, we observed minimal differences among case women with information on methylation status and all LIBCSP cases. Also, classification of methylation status is not universally defined and our cutoff of 4% may not be biologically relevant for all the genes assessed. We used a panel of a priori genes, [14] and thus, we cannot discount other methylation sites which could be relevant to reproductive characteristics and breast cancer. Given that biological significance is often 5′—C—phosphate—G—3′ (CpG) or region-specific, our lack of expected results for *CDH1* and *RASSF1A* may be related to not hitting on the ‘right’ CpGs for these genes. We did not adjust for multiple comparisons, because of the limited number of genes considered and because associations were driven by biologically plausible hypotheses. However, we recognize that some of these associations may be due to chance given the low prevalence of methylation, in some instances, and imprecise estimates. Finally, a potential limitation of the study is that women are now having their children at an older age than the mean/median experienced by the LIBCSP women. However, we anticipate that the biologic mechanisms underlying the association between late age at first birth, methylation, and cancer would be consistent despite a shift in age distribution. Our findings help to provide proof of principle for our novel hypothesis, and future studies could examine this issue with points further along a potential dose response curve.

## Conclusions

Among a large population-based sample, age at first birth and lactation were differentially associated with breast cancer based on the DNA methylation status of the tumor. While our results require confirmation in larger studies with robust gene coverage, they suggest that reproductive history may associate with gene promotors implicated in breast carcinogenesis which could be biomarkers of risk or molecular targets for prevention.

## Supplementary information

Additional file: **Table S1** Age-adjusted odds ratios (ORs) and 95% confidence intervals (CIs) for the association between age at menarche and ER + PR+ breast cancer (vs. all other ER + PR-, ER-PR+, ER-PR-) considering effect modification by gene specific promoter methylation, Long Island Breast Cancer Study. **Table S2** Age-adjusted odds ratios (ORs) and 95% confidence intervals (CIs) for the association between age at first birth and ER + PR+ breast cancer (vs. all other ER + PR-, ER-PR+, ER-PR-) considering effect modification by gene specific promoter methylation, Long Island Breast Cancer Study. **Table S3** Age-adjusted odds ratios (ORs) and 95% confidence intervals (CIs) for the association between parity and ER + PR+ breast cancer (vs. all other ER + PR-, ER-PR+, ER-PR-) considering effect modification by gene specific promoter methylation, Long Island Breast Cancer Study. **Table S4** Age-adjusted odds ratios (ORs) and 95% confidence intervals (CIs) for the association between parity and ER + PR+ breast cancer (vs. all other ER + PR-, ER-PR+, ER-PR-) considering effect modification by gene specific promoter methylation, Long Island Breast Cancer Study. (DOCX 67 kb)

## Data Availability

The datasets used and/or analysed during the current study are available from the study PI, Dr. Marilie Gammon gammon@unc.edu, on reasonable request.

## Abbreviations

*APC*: Adenomatous polyposis coli
*BRCA1*: Breast cancer 1, early onset
*CCND2*: Cyclin D2
*CDH1*: E-Cadherin
*CDKN2A*: Cyclin-dependent kinase inhibitor 2A
CI: Confidence interval
CpG: 5′—C—phosphate—G—3′
DAG: Directed acyclic graph
*DAPK1*: Death-associated protein kinase 1
EMT: Epithelial-mesenchymal transition
ER: Estrogen receptor
*ESR1*: Estrogen receptor 1
*GSTP1*: Glutathione S-transferase pi 1
HER2: Human epidermal growth factor receptor 2
*HIN1*: Secretoglobin, family 3A, member 1
LIBCSP: Long Island Breast Cancer Study Project
MSP: Methylation specific
NY: New York
OR: Odds ratio
PCR: Polymerase chain reaction
*PGR*: Progesterone gene receptor
PR: Progesterone receptor
*RARβ*: Retinoic acid receptor beta
*RASSF1A*: Ras association domain family member 1
TCGA: The Cancer Genome Atlas
*TWIST1*: Twist homolog 1
