# Supplementary material for: Reproductive characteristics are associated with gene-specific promoter methylation status in breast cancer
Source: BMC Cancer. 2019 Sep 18;19:926. doi: 10.1186/s12885-019-6120-4 (PMC6749688; doi:10.1186/s12885-019-6120-4)
Supplement: Supplementary file 1 — Table S1 Age-adjusted odds ratios (ORs) and 95% confidence intervals (CIs) for the association between age at menarche and ER + PR+ breast cancer (vs. all other ER + PR-, ER-PR+, ER-PR-) considering effect modification by gene specific promoter methylation, Long Island Breast Cancer Study. Table S2 Age-adjusted odds ratios (ORs) and 95% confidence intervals (CIs) for the association between age at first birth and ER + PR+ breast cancer (vs. all other ER + PR-, ER-PR+, ER-PR-) considering effect modification by gene specific promoter methylation, Long Island Breast Cancer Study. Table S3 Age-adjusted odds ratios (ORs) and 95% confidence intervals (CIs) for the association between parity and ER + PR+ breast cancer (vs. all other ER + PR-, ER-PR+, ER-PR-) considering effect modification by gene specific promoter methylation, Long Island Breast Cancer Study. Table S4 Age-adjusted odds ratios (ORs) and 95% confidence intervals (CIs) for the association between parity and ER + PR+ breast cancer (vs. all other ER + PR-, ER-PR+, ER-PR-) considering effect modification by gene specific promoter methylation, Long Island Breast Cancer Study. (DOCX 67 kb) [file 12885_2019_6120_MOESM1_ESM.docx]

| **Table S1.** Age-adjusted odds ratios (ORs) and 95% confidence intervals (CIs) for the association between age at menarche and ER+PR+ breast cancer (vs. all other ER+PR-, ER-PR+, ER-PR-) considering effect modification by gene specific promoter methylation, Long Island Breast Cancer Study. | | | | | | | | | | | |
| --- | --- | --- | --- | --- | --- | --- | --- | --- | --- | --- | --- |
|  |  | **All Breast Cancer Cases** | | | **Methylated breast tumor** | | | **Unmethylated breast tumor** | | |  |
| ***Gene promoter*** | **Age at Menarche** | **ER+PR+/all others** | **OR** | **95% CI** | **ER+PR+/all others** | **OR** | **95% CI** | **ER+PR+/all others** | **OR** | **95% CI** | ***p for interaction*** |
| ***APC*** |  |  |  |  |  |  |  |  |  |  |  |
|  | ***>12 years*** | 215/116 | 1.00 | reference | 101/52 | 1.00 | reference | 114/64 | 1.00 | reference | *0.68* |
|  | **≤*12 years*** | 135/114 | 0.64 | 0.46-0.89 | 68/59 | 0.59 | 0.36-0.95 | 67/55 | 0.68 | 0.43-1.09 |  |
| ***BRCA1*** |  |  |  |  |  |  |  |  |  |  |  |
|  | ***>12 years*** | 227/125 | 1.00 | reference | 146/73 | 1.00 | reference | 81/52 | 1.00 | reference | *0.17* |
|  | **≤*12 years*** | 145/119 | 0.67 | 0.49-0.93 | 78/70 | 0.55 | 0.36-0.85 | 67/49 | 0.87 | 0.53-1.45 |  |
| ***CDH1*** |  |  |  |  |  |  |  |  |  |  |  |
|  | ***>12 years*** | 203/115 | 1.00 | reference | 11/9 | 1.00 | reference | 192/106 | 1.00 | reference | *0.46* |
|  | **≤*12 years*** | 131/108 | 0.69 | 0.49-0.97 | 5/10 | 0.41 | 0.10-1.63 | 126/98 | 0.71 | 0.50-1.01 |  |
| ***CCND2*** |  |  |  |  |  |  |  |  |  |  |  |
|  | ***>12 years*** | 203/115 | 1.00 | reference | 41/25 | 1.00 | reference | 162/90 | 1.00 | reference | *0.81* |
|  | **≤*12 years*** | 131/108 | 0.69 | 0.49-0.97 | 25/24 | 0.64 | 0.30-1.33 | 106/84 | 0.7 | 0.48-1.03 |  |
| ***DAPK*** |  |  |  |  |  |  |  |  |  |  |  |
|  | ***>12 years*** | 203/115 | 1.00 | reference | 35/13 | 1.00 | reference | 168/102 | 1.00 | reference | *0.48* |
|  | **≤*12 years*** | 141/108 | 0.68 | 0.49-0.96 | 22/16 | 0.44 | 0.17-1.14 | 109/92 | 0.72 | 0.50-1.04 |  |
| ***ESR1*** |  |  |  |  |  |  |  |  |  |  |  |
|  | ***>12 years*** | 225/125 | 1.00 | reference | 101/47 | 1.00 | reference | 124/78 | 1.00 | reference | *0.08* |
|  | **≤*12 years*** | 143/118 | 0.67 | 0.48-0.93 | 61/59 | 0.48 | 0.29-0.79 | 82/59 | 0.87 | 0.56-1.35 |  |
| ***GSTP1*** |  |  |  |  |  |  |  |  |  |  |  |
|  | ***>12 years*** | 203/115 | 1.00 | reference | 56/31 | 1.00 | reference | 147/84 | 1.00 | reference | *0.25* |
|  | **≤*12 years*** | 131/108 | 0.69 | 0.49-0.97 | 34/37 | 0.51 | 0.27-0.96 | 97/71 | 0.78 | 0.52-1.18 |  |
| ***HIN*** |  |  |  |  |  |  |  |  |  |  |  |
|  | ***>12 years*** | 203/115 | 1.00 | reference | 136/59 | 1.00 | reference | 57/56 | 1.00 | reference | *0.58* |
|  | **≤*12 years*** | 131/108 | 0.69 | 0.49-0.97 | 85/58 | 0.63 | 0.40-1.00 | 46/50 | 0.77 | 0.45-1.32 |  |
| ***P16*** |  |  |  |  |  |  |  |  |  |  |  |
|  | ***>12 years*** | 209/116 | 1.00 | reference | 5/4 | 1.00 | reference | 204/112 | 1.00 | reference | *0.71* |
|  | **≤*12 years*** | 130/114 | 0.64 | 0.46-0.90 | 4/7 | not estimated^a^ | | 126/107 | 0.65 | 0.46-0.91 |  |
| ***PR*** |  |  |  |  |  |  |  |  |  |  |  |
|  | ***>12 years*** | 227/125 | 1.00 | reference | 21/16 | 1.00 | reference | 206/109 | 1.00 | reference | *0.69* |
|  | **≤*12 years*** | 145/119 | 0.68 | 0.49-0.94 | 18/17 | 0.81 | 0.32-2.07 | 127/102 | 0.66 | 0.46-0.93 |  |
| ***RARB*** |  |  |  |  |  |  |  |  |  |  |  |
|  | ***>12 years*** | 203/115 | 1.00 | reference | 55/38 | 1.00 | reference | 148/77 | 1.00 | reference | *0.19* |
|  | **≤*12 years*** | 131/108 | 0.67 | 0.48-0.95 | 25/37 | 0.46 | 0.24-0.89 | 107/71 | 0.78 | 0.52-1.17 |  |
| ***RASSF1A*** |  |  |  |  |  |  |  |  |  |  |  |
|  | ***>12 years*** | 203/115 | 1.00 | reference | 185/89 | 1.00 | reference | 18/26 | 1.00 | reference | *0.04* |
|  | **≤*12 years*** | 131/108 | 0.69 | 0.49-0.97 | 112/91 | 0.59 | 0.40-0.86 | 19/17 | 1.64 | 0.67-3.99 |  |
| ***TWIST1*** |  |  |  |  |  |  |  |  |  |  |  |
|  | ***>12 years*** | 203/115 | 1.00 | reference | 30/18 | 1.00 | reference | 173/97 | 1.00 | reference | *0.28* |
|  | **≤*12 years*** | 131/108 | 0.69 | 0.49-0.98 | 18/24 | 0.46 | 0.20-1.07 | 113/84 | 0.75 | 0.52-1.10 |  |
| ^a^ Point estimate was not calculated because cell sizes less than five | | | | | |  |  |  |  |  |  |

| **Table S2**: Age-adjusted odds ratios (ORs) and 95% confidence intervals (CIs) for the association between age at first birth and ER+PR+ breast cancer (vs. all other ER+PR-, ER-PR+, ER-PR-) considering effect modification by gene specific promoter methylation, Long Island Breast Cancer Study. | | | | | | | | | | | |
| --- | --- | --- | --- | --- | --- | --- | --- | --- | --- | --- | --- |
|  |  | **All Breast Cancer Cases** | | | **Methylated breast tumor** | | | **Unmethylated breast tumor** | | |  |
| **Gene Promoter** | **Age at First Birth** | **ER+PR+/all others** | **OR** | **95% CI** | **ER+PR+/all others** | **OR** | **95% CI** | **ER+PR+/all others** | **OR** | **95% CI** | ***p for interaction*** |
| ***APC*** | **≤*23 years*** | 93/75 | 1.00 | reference | 39/38 | 1.00 | reference | 54/37 | 1.00 | reference |  |
|  | ***23-27 years*** | 106/69 | 1.17 | 0.94-1.46 | 58/25 | 1.14 | 0.83-1.57 | 48/44 | 1.21 | 0.89-4.23 |  |
|  | **≥27 years** | 103/61 | 1.38 | 0.88-2.14 | 49/37 | 1.31 | 0.70-2.47 | 54/24 | 1.47 | 0.79-2.72 | *0.77* |
| ***BRCA1*** |  |  |  |  |  |  |  |  |  |  |  |
|  | **≤*23 years*** | 100/78 | 1.00 | reference | 64/39 | 1.00 | reference | 36/39 | 1.00 | reference |  |
|  | ***23-27 years*** | 110/72 | 1.14 | 0.92-1.40 | 60/42 | 0.94 | 0.71-1.23 | 50/30 | 1.53 | 1.08-2.15 | *0.03* |
|  | **≥27 years** | 112/68 | 1.29 | 0.84-1.97 | 66/26 | 0.88 | 0.51-1.51 | 46/22 | 2.34 | 1.18-4.64 |  |
| ***CDH1*** |  |  |  |  |  |  |  |  |  |  |  |
|  | **≤*23 years*** | 96/74 | 1.00 | reference | 9/7 | 1.00 | reference | 87/67 | 1.00 | reference |  |
|  | ***23-27 years*** | 96/63 | 1.13 | 0.90-1.41 | 2/7 | not estimated^a^ | | 94/56 | 1.13 | 0.90-1.41 | *0.95* |
|  | **≥27 years** | 103/62 | 1.27 | 0.82-1.98 | 16/2 | not estimated | | 98/60 | 1.27 | 0.80-2.00 |  |
| ***CCND2*** |  |  |  |  |  |  |  |  |  |  |  |
|  | **≤*23 years*** | 96/74 | 1.00 | reference | 21/17 | 1.00 | reference | 75/57 | 1.00 | reference |  |
|  | ***23-27 years*** | 96/63 | 1.14 | 0.91-1.42 | 22/12 | 1.08 | 0.67-1.72 | 74/51 | 1.17 | 0.91-1.49 |  |
|  | **≥27 years** | 103/62 | 1.3 | 0.84-2.01 | 21/16 | 1.16 | 0.45-2.96 | 82/46 | 1.36 | 0.83-2.24 | *0.70* |
| ***DAPK*** |  |  |  |  |  |  |  |  |  |  |  |
|  | **≤*23 years*** | 96/74 | 1.00 | reference | 20/11 | 1.00 | reference | 76/63 | 1.00 | reference |  |
|  | ***23-27 years*** | 96/63 | 1.16 | 0.93-1.45 | 18/9 | 1.55 | 0.81-2.99 | 78/54 | 1.12 | 0.88-1.42 |  |
|  | **≥27 years** | 103/62 | 1.34 | 0.87-2.09 | 17/4 | 2.4 | 0.65-8.91 | 86/58 | 1.25 | 0.78-2.01 | *0.42* |
| ***ESR1*** |  |  |  |  |  |  |  |  |  |  |  |
|  | **≤*23 years*** | 98/78 | 1.00 | reference | 45/31 |  |  | 53/47 |  |  |  |
|  | ***23-27 years*** | 110/71 | 1.14 | 0.92-1.41 | 49/34 | 1.02 | 0.73-1.41 | 61/37 | 1.22 | 0.92-1.62 |  |
|  | **≥27 years** | 110/68 | 1.29 | 0.84-1.98 | 36/30 | 1.03 | 0.54-1.98 | 64/38 | 1.5 | 0.85-2.64 | *0.43* |
| ***GSTP1*** |  |  |  |  |  |  |  |  |  |  |  |
|  | **≤*23 years*** | 96/74 | 1.00 | reference | 28/25 | 1.00 | reference | 68/49 | 1.00 | reference |  |
|  | ***23-27 years*** | 96/63 | 1.14 | 0.91-1.42 | 25/19 | 1.37 | 0.91-2.07 | 71/44 | 1.05 | 0.81-1.37 |  |
|  | **≥27 years** | 103/62 | 1.3 | 0.83-2.01 | 30/14 | 1.88 | 0.83-4.29 | 73/48 | 1.11 | 0.66-1.87 | *0.29* |
| ***HIN*** |  |  |  |  |  |  |  |  |  |  |  |
|  | **≤*23 years*** | 96/74 | 1.00 | reference | 63/43 | 1.00 | reference | 33/31 | 1.00 | reference |  |
|  | ***23-27 years*** | 96/63 | 1.16 | 0.93-1.44 | 61/33 | 1.32 | 0.98-1.78 | 35/30 | 0.96 | 0.69-1.35 |  |
|  | **≥27 years** | 103/62 | 1.34 | 0.86-2.09 | 68/27 | 1.75 | 0.97-3.16 | 35/35 | 0.93 | 0.47-1.84 | *0.17* |
| ***P16*** |  |  |  |  |  |  |  |  |  |  |  |
|  | **≤*23 years*** | 89/75 | 1.00 | reference | 5/4 | not estimated | | 84/71 | 1.00 | reference | *--* |
|  | ***23-27 years*** | 101/69 | 1.18 | 0.95-1.47 | 0/4 | not estimated | | 101/65 | 1.18 | 0.94-1.48 |  |
|  | **≥27 years** | 101/61 | 1.39 | 0.89-2.17 | 4/2 | not estimated | | 97/59 | 1.4 | 0.89-2.20 |  |
| ***PR*** |  |  |  |  |  |  |  |  |  |  |  |
|  | **≤*23 years*** | 100/78 | 1.00 | reference | 6/10 | 1.00 | reference | 94/68 | 1.00 | reference |  |
|  | ***23-27 years*** | 110/72 | 1.15 | 0.93-1.43 | 13/6 | 1.07 | 0.58-1.99 | 97/66 | 1.16 | 0.93-1.46 |  |
|  | **≥27 years** | 112/68 | 1.33 | 0.87-2.04 | 13/15 | 1.16 | 0.34-3.94 | 99/53 | 1.36 | 0.86-2.14 | *0.82* |
| ***RARB*** |  |  |  |  |  |  |  |  |  |  |  |
|  | **≤*23 years*** | 96/74 | 1.00 | reference | 18/27 | 1.00 | reference | 78/47 | 1.00 | reference |  |
|  | ***23-27 years*** | 96/63 | 1.14 | 0.92-1.43 | 27/19 | 1.47 | 0.96-2.26 | 69/44 | 1.05 | 0.81-1.36 |  |
|  | **≥27 years** | 103/62 | 1.31 | 0.84-2.04 | 26/20 | 2.17 | 0.92-5.11 | 77/42 | 1.1 | 0.66-1.86 | *0.23* |
| ***RASSF1A*** |  |  |  |  |  |  |  |  |  |  |  |
|  | **≤*23 years*** | 96/74 | 1.00 | reference | 84/58 | 1.00 | reference | 12/16 | 1.00 | reference |  |
|  | ***23-27 years*** | 96/63 | 1.13 | 0.91-1.41 | 81/53 | 1.16 | 0.91-1.48 | 15/10 | 1.02 | 0.58-1.80 |  |
|  | **≥27 years** | 103/62 | 1.28 | 0.82-1.98 | 94/50 | 1.35 | 0.83-2.18 | 9/12 | 1.04 | 0.34-3.22 | *0.70* |
| ***TWIST1*** |  |  |  |  |  |  |  |  |  |  |  |
|  | **≤*23 years*** | 96/74 | 1.00 | reference | 18/17 | 1.00 | reference | 78/57 | 1.00 | reference |  |
|  | ***23-27 years*** | 96/63 | 1.12 | 0.90-1.40 | 13/12 | 1.31 | 0.71-2.40 | 83/51 | 1.11 | 0.88-1.41 |  |
|  | **≥27 years** | 103/62 | 1.26 | 0.81-1.97 | 10/7 | 1.71 | 0.51-5.77 | 93/55 | 1.24 | 0.77-2.00 | *0.85* |
| ^a^ Point estimate was not calculated because cell sizes less than five | | | | | |  |  |  |  |  |  |

| **Table S3.** Age-adjusted odds ratios (ORs) and 95% confidence intervals (CIs) for the association between parity and ER+PR+ breast cancer (vs. all other ER+PR-, ER-PR+, ER-PR-) considering effect modification by gene specific promoter methylation, Long Island Breast Cancer Study. | | | | | | | | | | | |
| --- | --- | --- | --- | --- | --- | --- | --- | --- | --- | --- | --- |
|  |  | **All Breast Cancer Cases** | | | **Methylated breast tumor** | | | **Unmethylated breast tumor** | | |  |
| ***Gene promoter*** | **Lactation** | **ER+PR+/all others** | **OR** | **95% CI** | **ER+PR+/all others** | **OR** | **95% CI** | **ER+PR+/all others** | **OR** | **95% CI** | **p for interaction** |
| ***APC*** |  |  |  |  |  |  |  |  |  |  |  |
|  | ***Any lactation*** | 120/72 | 1.00 | reference | 48/34 | 1.00 | reference | 72/38 | 1.00 | reference | 0.22 |
|  | **No lactation** | 182/133 | 0.82 | 0.57-1.20 | 98/66 | 1.06 | 0.62-1.82 | 84/67 | 0.66 | 0.40-1.10 |  |
| ***BRCA1*** |  |  |  |  |  |  |  |  |  |  |  |
|  | ***Any lactation*** | 125/78 | 1.00 | reference | 76/48 | 1.00 | reference | 49/30 | 1.00 | reference | 0.80 |
|  | **No lactation** | 197/140 | 0.88 | 0.62-1.26 | 114/79 | 0.93 | 0.58-1.48 | 83/61 | 0.83 | 0.48-1.46 |  |
| ***CDH1*** |  |  |  |  |  |  |  |  |  |  |  |
|  | ***Any lactation*** | 117/69 | 1.00 | reference | 9/6 | 1.00 | reference | 108/63 | 1.00 | reference | 0.45 |
|  | **No lactation** | 178/130 | 0.8 | 0.55-1.17 | 7/10 | 0.45 | 0.11-1.90 | 171/120 | 0.84 | 0.57-1.23 |  |
| ***CCND2*** |  |  |  |  |  |  |  |  |  |  |  |
|  | ***Any lactation*** | 117/69 | 1.00 | reference | 29/14 | 1.00 | reference | 88/55 | 1.00 | reference | 0.24 |
|  | **No lactation** | 178/130 | 0.81 | 0.56-1.18 | 35/31 | 0.5 | 0.22-1.14 | 143/99 | 0.91 | 0.60-1.40 |  |
| ***DAPK*** |  |  |  |  |  |  |  |  |  |  |  |
|  | ***Any lactation*** | 117/69 | 1.00 | reference | 25/10 | 1.00 | reference | 92/59 | 1.00 | reference | 0.89 |
|  | **No lactation** | 178/130 | 0.83 | 0.57-1.21 | 30/14 | 1 | 0.37-2.73 | 148/116 | 0.82 | 0.55-1.23 |  |
| ***ESR1*** |  |  |  |  |  |  |  |  |  |  |  |
|  | ***Any lactation*** | 123/77 | 1.00 | reference | 54/31 | 1.00 | reference | 69/46 | 1.00 | reference | 0.55 |
|  | **No lactation** | 195/140 | 0.87 | 0.61-1.25 | 86/54 | 0.77 | 0.45-1.34 | 109/76 | 1 | 0.62-1.61 |  |
| ***GSTP1*** |  |  |  |  |  |  |  |  |  |  |  |
|  | ***Any lactation*** | 117/69 | 1.00 | reference | 34/20 | 1.00 | reference | 83/49 | 1.00 | reference | 0.85 |
|  | **No lactation** | 178/130 | 0.81 | 0.56-1.18 | 49/38 | 0.76 | 0.38-1.52 | 129/92 | 0.83 | 0.54-1.30 |  |
| ***HIN*** |  |  |  |  |  |  |  |  |  |  |  |
|  | ***Any lactation*** | 117/69 | 1.00 | reference | 80/39 | 1.00 | reference | 37/30 | 1.00 | reference | 0.88 |
|  | **No lactation** | 178/130 | 0.84 | 0.58-1.23 | 112/64 | 0.86 | 0.53-1.41 | 66/66 | 0.81 | 0.45-1.47 |  |
| ***P16*** |  |  |  |  |  |  |  |  |  |  |  |
|  | ***Any lactation*** | 116/72 | 1.00 | reference | 3/6 | not estimated^a^ | | 113/66 | 1.00 | reference | -- |
|  | **No lactation** | 175/133 | 0.82 | 0.56-1.18 | 6/4 | not estimated | | 169/129 | 0.77 | 0.53-1.13 |  |
| ***PR*** |  |  |  |  |  |  |  |  |  |  |  |
|  | ***Any lactation*** | 125/78 | 1.00 | reference | 16/13 | 1.00 | reference | 109/65 | 1.00 | reference | 0.73 |
|  | **No lactation** | 197/140 | 0.87 | 0.61-1.24 | 16/18 | 0.74 | 0.27-2.06 | 181/122 | 0.89 | 0.60-1.30 |  |
| ***RARB*** |  |  |  |  |  |  |  |  |  |  |  |
|  | ***Any lactation*** | 117/69 | 1.00 | reference | 35/22 | 1.00 | reference | 82/47 | 1.00 | reference | 0.16 |
|  | **No lactation** | 178/130 | 0.79 | 0.55-1.16 | 26/44 | 0.54 | 0.27-1.08 | 142/86 | 0.95 | 0.60-1.48 |  |
| ***RASSF1A*** | |  |  |  |  |  |  |  |  |  |  |
|  | ***Any lactation*** | 117/69 | 1.00 | reference | 108/58 | 1.00 | reference | 9/11 | 1.00 | reference | 0.40 |
|  | **No lactation** | 178/130 | 0.84 | 0.58-1.23 | 151/103 | 0.79 | 0.53-1.19 | 27/27 | 1.28 | 0.45-3.59 |  |
| ***TWIST1*** |  |  |  |  |  |  |  |  |  |  |  |
|  | ***Any lactation*** | 117/69 | 1.00 | reference | 15/13 | 1.00 | reference | 102/56 | 1.00 | reference | 0.66 |
|  | **No lactation** | 178/130 | 0.81 | 0.56-1.19 | 26/23 | 1.01 | 0.39-2.60 | 152/107 | 0.78 | 0.52-1.18 |  |
| ^a^ Point estimate was not calculated because cell sizes less than five | | | | | |  |  |  |  |  |  |

| **Table S4.**  Age-adjusted odds ratios (ORs) and 95% confidence intervals (CIs) for the association between parity and ER+PR+ breast cancer (vs. all other ER+PR-, ER-PR+, ER-PR-) considering effect modification by gene specific promoter methylation, Long Island Breast Cancer Study. | | | | | | | | | | | |
| --- | --- | --- | --- | --- | --- | --- | --- | --- | --- | --- | --- |
|  |  | **All Breast Cancer Cases** | | | **Methylated breast tumor** | | | **Unmethylated breast tumor** | | |  |
| ***Gene promoter*** | **Parity** | **ER+PR+/all others** | **OR** | **95% CI** | **ER+PR+/all others** | **OR** | **95% CI** | **ER+PR+/all others** | **OR** | **95% CI** | ***p for interaction*** |
| ***APC*** |  |  |  |  |  |  |  |  |  |  |  |
|  | ***Parous*** | 302/205 | 1.00 | reference | 146/100 | 1.00 | reference | 156/105 | 1.00 | reference | *0.59* |
|  | **Nulliparous** | 50/31 | 1.09 | 0.67-1.76 | 24/13 | 1.23 | 0.60-2.54 | 26/18 | 1.01 | 0.52-1.95 |  |
| ***BRCA1*** |  |  |  |  |  |  |  |  |  |  |  |
|  | ***Parous*** | 322/218 | 1.00 | reference | 190/127 | 1.00 | reference | 132/91 | 1.00 | reference | *0.49* |
|  | **Nulliparous** | 53/32 | 1.11 | 0.69-1.79 | 36/19 | 1.25 | 0.69-2.28 | 17/13 | 0.94 | 0.43-2.04 |  |
| ***CDH1*** |  |  |  |  |  |  |  |  |  |  |  |
|  | ***Parous*** | 295/199 | 1.00 | reference | 16/16 | 1.00 | reference | 279/183 | 1.00 | reference | *--* |
|  | **Nulliparous** | 42/28 | 0.99 | 0.59-1.66 | 1/3 | not estimated^a^ | | 41/25 | 1.06 | 0.62-1.80 |  |
| ***CCND2*** |  |  |  |  |  |  |  |  |  |  |  |
|  | ***Parous*** | 295/199 | 1.00 | reference | 64/45 | 1.00 | reference | 231/154 | 1.00 | reference | *--* |
|  | **Nulliparous** | 42/28 | 0.99 | 0.59-1.65 | 4/5 | not estimated | | 39/23 | 1.12 | 0.64-1.95 |  |
| ***DAPK*** |  |  |  |  |  |  |  |  |  |  |  |
|  | ***Parous*** | 295/199 | 1.00 | reference | 55/24 | 1.00 | reference | 240/175 | 1.00 | reference | *--* |
|  | **Nulliparous** | 42/28 | 1.01 | 0.60-1.69 | 3/5 | not estimated | | 39/23 | 1.21 | 0.70-2.11 |  |
| ***ESR1*** |  |  |  |  |  |  |  |  |  |  |  |
|  | ***Parous*** | 318/217 | 1.00 | reference | 140/95 | 1.00 | reference | 178/122 | 1.00 | reference | *0.91* |
|  | **Nulliparous** | 53/32 | 1.13 | 0.70-1.81 | 24/14 | 1.17 | 0.57-2.37 | 29/18 | 1.08 | 0.57-2.05 |  |
| ***GSTP1*** |  |  |  |  |  |  |  |  |  |  |  |
|  | ***Parous*** | 295/199 | 1.00 | reference | 83/58 | 1.00 | reference | 212/141 | 1.00 | reference | *0.06* |
|  | **Nulliparous** | 42/28 | 0.99 | 0.59-1.65 | 7/11 | 0.44 | 0.16-1.21 | 35/17 | 1.35 | 0.72-2.50 |  |
| ***HIN*** |  |  |  |  |  |  |  |  |  |  |  |
|  | ***Parous*** | 295/199 | 1.00 | reference | 192/103 | 1.00 | reference | 103/96 | 1.00 | reference | *0.55* |
|  | **Nulliparous** | 42/28 | 0.94 | 0.56-1.59 | 32/16 | 1.06 | 0.56-2.03 | 10/12 | 0.76 | 0.31-1.84 |  |
| ***P16*** |  |  |  |  |  |  |  |  |  |  |  |
|  | ***Parous*** | 291/205 | 1.00 | reference | 9/10 | 1.00 | reference | 282/195 | 1.00 | reference | *--* |
|  | **Nulliparous** | 50/31 | 1.11 | 0.68-1.80 | 0/1 | not estimated | | 50/30 | 1.14 | 0.70-1.87 |  |
| ***PR*** |  |  |  |  |  |  |  |  |  |  |  |
|  | ***Parous*** | 322/218 | 1.00 | reference | 32/31 | 1.00 | reference | 290/187 | 1.00 | reference | *--* |
|  | **Nulliparous** | 53/32 | 1.12 | 0.70-1.80 | 7/4 | not estimated | | 46/28 | 1.05 | 0.63-1.75 |  |
| ***RARB*** |  |  |  |  |  |  |  |  |  |  |  |
|  | ***Parous*** | 295/199 | 1.00 | reference | 71/66 | 1.00 | reference | 224/133 | 1.00 | reference | *0.97* |
|  | **Nulliparous** | 42/28 | 1.01 | 0.60-1.69 | 11/10 | 1.02 | 0.41-2.57 | 31/18 | 1.02 | 0.55-1.91 |  |
| ***RASSF1A*** | |  |  |  |  |  |  |  |  |  |  |
|  | ***Parous*** | 295/199 | 1.00 | reference | 259/161 | 1.00 | reference | 36/38 | 1.00 | reference | *--* |
|  | **Nulliparous** | 42/28 | 0.95 | 0.57-1.59 | 41/23 | 1.09 | 0.63-1.89 | 1/5 | not estimated | |  |
| ***TWIST1*** |  |  |  |  |  |  |  |  |  |  |  |
|  | ***Parous*** | 295/199 | 1.00 | reference | 41/37 | 1.00 | reference | 254/163 | 1.00 | reference | *0.77* |
|  | **Nulliparous** | 42/28 | 1.1 | 0.61-1.70 | 7/7 | 0.85 | 0.27-2.67 | 35/21 | 1.06 | 0.59-1.90 |  |
| ^a^ Point estimate was not calculated because cell sizes less than five | | | | | | |  |  |  |  |  |
